# Polymer-Coated Covalent Organic Frameworks as Porous Liquids for Gas Storage

**DOI:** 10.1021/acs.chemmater.3c02828

**Published:** 2024-01-19

**Authors:** Rachel E. Mow, Glory A. Russell-Parks, Grace E. B. Redwine, Brittney E. Petel, Thomas Gennett, Wade A. Braunecker

**Affiliations:** † Materials Science Program, Colorado School of Mines, Golden, Colorado 80401, United States; ‡ Department of Chemistry, Colorado School of Mines, 1012 14th Street, Golden, Colorado 80401, United States; § Chemistry and Nanoscience Center, National Renewable Energy Laboratory, 15013 Denver West Pkwy, Golden, Colorado 80401, United States; ∥ Catalytic Carbon Transformation and Scale-Up Center, National Renewable Energy Laboratory, 15013 Denver West Pkwy, Golden, Colorado 80401, United States

## Abstract

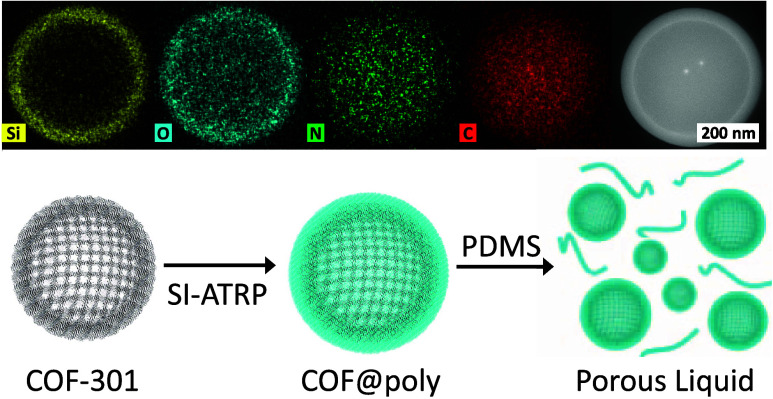

Several synthetic methods have recently emerged to develop high-surface-area solid-state organic framework-based materials into free-flowing liquids with permanent porosity. The fluidity of these porous liquid (PL) materials provides them with advantages in certain storage and transport processes. However, most framework-based materials necessitate the use of cryogenic temperatures to store weakly bound gases such as H_2_, temperatures where PLs lose their fluidity. Covalent organic framework (COF)-based PLs that could reversibly form stable complexes with H_2_ near ambient temperatures would represent a promising development for gas storage and transport applications. We report here the development, characterization, and evaluation of a material with these remarkable characteristics based on Cu(I)-loaded COF colloids. Our synthetic strategy required tailoring conditions for growing robust coatings of poly(dimethylsiloxane)-methacrylate (PDMS-MA) around COF colloids using atom transfer radical polymerization (ATRP). We demonstrate exquisite control over the coating thickness on the colloidal COF, quantified by transmission electron microscopy and dynamic light scattering. The coated COF material was then suspended in a liquid polymer matrix to make a PL. CO_2_ isotherms confirmed that the coating preserved the general porosity of the COF in the free-flowing liquid, while CO sorption measurements using diffuse reflectance infrared Fourier transform spectroscopy (DRIFTS) confirmed the preservation of Cu(I) coordination sites. We then evaluated the gas sorption phenomenon in the Cu(I)–COF-based PLs using DRIFTS and temperature-programmed desorption measurements. In addition to confirming that H_2_ transport is possible at or near mild refrigeration temperatures with these materials, our observations indicate that H_2_ diffusion is significantly influenced by the glass-transition temperature of both the coating and the liquid matrix. The latter result underscores an additional potential advantage of PLs in tailoring gas diffusion and storage temperatures through the coating composition.

## 1 Introduction

Solid-state porous organic materials have become promising platforms for a wide range of research applications, spanning disciplines that include biomedicine,^1,2^ energy storage,^3,4^ catalysis,^5−7^ gas storage and separations,^8−10^ and numerous other energy-related applications.^11^ Covalent organic frameworks (COFs) constitute one subset of these materials; composed of lightweight elements and defined by periodic structures with rigid covalent bonds, the highly tunable pore sizes, shapes, and functionalities in COFs give these materials distinct advantages over porous inorganic zeolites and silicates when it comes to tailoring a material for a specific application.^12,13^ With respect to gas storage and separations, various pore engineering strategies have recently produced COFs for advanced CO_2_ sequestration,^14,15^ methane storage,^16,17^ ethylene/ethane separation,^18,19^ etc. However, COF powders generally suffer from relatively weak interactions with gases like H_2_, which limits the applicability of these materials for H_2_ storage and transport.^20^ As such, new synthetic design strategies are necessary to further improve COF-based solutions in these fields.

One synthetic modification strategy garnering recent attention is the development of solid framework-based materials into “porous liquids” (PLs).^21,22^ This unique class of materials possesses both permanent porosity and fluidity and has already shown promise for various carbon capture applications^23,24^ and hydrocarbon separations.^25,26^ The fluidity of PLs can also improve heat transfer over their solid-state counterparts, reducing the cost and energy associated with regeneration.^27^ PLs further offer a unique opportunity to incorporate high-capacity, selective materials as drop-in replacements for industrial processes specifically designed to accommodate liquids. However, the viscosity of PLs can be a limitation for many of those applications, particularly PLs based on ionic liquids. While efforts have been made to develop PLs with lower viscosities,^28^ an additional challenge facing framework-based PLs intended for application with weakly physisorbing gases like H_2_ is that appreciable amounts of sorption only occur at cryogenic temperatures, conditions where the PL is frozen. To realize the full potential of PLs for H_2_ transport, they would need to remain free-flowing at temperatures at which gas sorption is significant.

As has been shown with metal organic framework (MOF) materials, the incorporation of open metal sites is one way to increase storage capacity, isosteric heat of adsorption, and the temperature that gas molecules desorb.^29−32^ Open metal sites were also recently incorporated into two-dimensional (2D) and three-dimensional (3D) COFs via postsynthetic modification of the framework. Stable complexes of Cu(I)–COFs with H_2_, ethylene (C_2_H_4_), and carbon monoxide (CO) near ambient temperatures were isolated and studied; remarkable values of H_2_ desorption enthalpies in these COFs were attributed to strong π-backbonding interactions.^33,34^

Here, we report the development of a pioneering COF-based PL that retains open Cu(I) sites capable of facilitating gas transport at mild refrigeration temperatures within a fluid matrix. We developed an innovative synthetic strategy tailored for growing robust polymeric coatings around COF colloids. The coating plays a pivotal role in several key features of the material, preventing the COF from permanent aggregation in a fluid matrix, preserving the material’s porosity in a PL, and enabling the material to withstand the high temperatures required to activate Cu(I) sites, facilitating subsequent CO and H_2_ bindings. Following a comprehensive characterization of these materials and a thorough evaluation of the feasibility of H_2_ transport near mild refrigeration temperatures in fluid matrices, we highlight an additional potential advantage of these materials by demonstrating how gas diffusion and storage temperatures can be finely tuned through the composition of the coating.

## 2 Experimental Section

### 2.1 General

Monomethacryloxypropyl-terminated poly(dimethylsiloxane), 10 cSt (PDMS-MA), was purchased from Gelest. Tetrakis(4-aminophenyl)methane was purchased from the Tokyo Chemical Industry. 2,5-Dihydroxy terephthalaldehyde was purchased from A-Chem Block. Poly(dimethylsiloxane), trimethylsiloxy-terminated (M.W. 6000) was purchased from Alfa Aesar. Both 1,4-dioxane and acetonitrile were purchased anhydrous from Sigma-Aldrich, further dried over 3 Å molecular sieves, and then passed through a 0.2 μm poly(tetrafluoroethylene) (PTFE) filter prior to use. All other chemicals were purchased and used as received from Sigma-Aldrich.

### 2.2 Synthetic Procedures

#### 2.2.1 Polymeric Initiator Synthesis

The polymeric initiator molecule [P(BIEM-*r*-VBA)] was synthesized via radical addition–fragmentation chain-transfer (RAFT) polymerization. A Schlenk flask was charged with 1.0 g (3.6 mmol) of 2-(2-bromoisobutyryloxy)ethyl methacrylate (BIEM), 63 mg (0.5 mmol) of 4-vinyl benzaldehyde (VBA), 31 mg (0.09 mmol) of 2-cyano-2-propyl dodecyltrithiocarbonate, 5 mg (0.03 mmol) of azobis(isobutyronitrile) (AIBN), and 1 mL of dimethylformamide. After three freeze–pump–thaw cycles, the reaction was heated at 60 °C for 40 h. P(BIEM-*r*-VBA) was purified by thrice precipitating into 20 mL of cold methanol, collecting by centrifugation (10,000 rpm, 10 min), and resuspending in 2 mL of tetrahydrofuran (THF). Finally, the product was dried on a Schlenk line vacuum overnight at 60 °C. ^1^H NMR spectroscopy was used to estimate the ratio of initiating (Br) to anchoring (aldehyde) sites as 94:6 (Figure S1).

#### 2.2.2 Atom Transfer Radical Polymerization (ATRP) of PDMS-MA

The following procedure describes optimized conditions for the model PDMS-MA brush polymerization. A Schlenk flask was loaded with a stir bar, 2 mg (0.02 mmol) of CuCl, 2.0 g (2 mmol) of PDMS-MA, 0.4 mL of dry acetonitrile, and 3.6 mL of dry 1,4-dioxane. After the heterogeneous mixture was sparged with N_2_ for 20 min, 5.4 μL (0.02 mmol) of N_2_-sparged tris[2-(dimethylamino)ethyl]amine (Me_6_TREN) was injected and the mixture was sonicated for 20 min to ensure full dissolution of the Cu catalyst. A T_0_ aliquot was taken, after which 15 μL of a 10 vol % stock solution of N_2_-sparged ethyl α-bromoisobutyrate initiator (EBriB, 0.01 mmol) in dioxane was injected. The polymerization was stirred at room temperature (rt) for 24 h, with aliquots taken systematically to monitor the polymer molecular weight increase (GPC) and conversion (NMR). ^1^H NMR indicated that a 70% conversion was achieved after 24 h (Figure S2).

#### 2.2.3 Colloidal COF Synthesis

Colloidal COF-301 was synthesized and purified according to a previously reported literature procedure.^8^ 80 mg (0.21 mmol) of tetrakis(4-aminophenyl) methane and 80 mg (0.48 mmol) of 2,5-dihydroxy terephthalaldehyde were added to a bomb flask and dissolved in 50 mL of dry acetonitrile. The mixture was sparged with N_2_ for 10 min, and then 10 μL (0.13 μmol) of trifluoroacetic acid was injected. After the mixture was sparged another 5 min, the reaction was stirred at 120 °C for 72 h. The COF colloid was purified with three cycles of centrifugation (14,000 rpm, 15 min), decanting, and resuspension/stirring in acetonitrile.

#### 2.2.4 COF-301@PDMS-MA Synthesis

P(BIEM-*r*-VBA) was first tethered to the COF-301 surface via a condensation reaction. Twenty mg of P(BIEM-*r*-VBA) was added to 100 mg of colloidal COF suspended in 30 mL of acetonitrile. Ten μL of trifluoroacetic acid was injected, and the reaction was stirred at 60 °C for 1 h. The P(BIEM-*r*-VBA)-coated COF was purified of acid catalyst and excess unreacted polymeric initiator with three centrifugation (14,000 rpm, 15 min), decanting, and resuspension cycles in acetonitrile. Surface-initiated ATRP (SI-ATRP) was then conducted to grow uniform PDMS-MA coatings around the COF colloids by adding 3 mg (0.03 mmol) of CuCl and 3 g (3 mmol) of PDMS-methacrylate (PDMS-MA) to a Schlenk flask. 100 mg of COF-301@initiator suspended in 0.6 mL of acetonitrile and 5.4 mL of dioxane was added to the solution. The mixture was sparged with N_2_ for 30 min and sonicated for 10 min. Eight μL (0.03 mmol) of N_2_-sparged Me_6_TREN was injected, and the reaction was stirred at rt for up to 24 h. The material was purified with five centrifuge (14,000 rpm, 15 min), decanting, and resuspension cycles in dichloromethane. The material was stored in dichloromethane. For cross-linked coatings, 10 mol % of cross-linker (ethylene glycol dimethacrylate) relative to the monomer was added along with the CuCl.

#### 2.2.5 Cu Loading into COF-301@PDMS-MA

100 mg portion of purified COF-301@PDMS-MA was stirred with 140 mg of Cu(II) formate in 50 mL of a 1:1 dichloromethane:methanol solution for 24 h at 40 °C. Note that if stirred at higher temperatures, we observed the deposition of metallic Cu throughout the sample, which we attributed to a partial reduction to Cu(I) that in turn disproportionated under these conditions. The Cu(II)–COF-301@PDMS-MA was purified with three centrifuge (14,000 rpm, 15 min), decanting, and resuspension cycles in methanol. Prior to activation, the Cu-loaded COF colloids were collected by centrifugation and dried overnight at rt on a Schlenk line vacuum to yield a brown powder. Cu(II) formate in the COF could then be efficiently converted to open Cu(I) sites by activation at 200 °C under ultrahigh vacuum for 2 h,^18,19^ yielding a black powder. The presence and location of Cu in the COF were confirmed by transmission electron microscopy-energy dispersive spectrometry (TEM-EDS) imaging (*vide infra*).

#### 2.2.6 Porous Liquid Synthesis

Prior to the synthesis of any porous liquids, both the Cu–COF-301@PDMS-MA and the PDMS (*M*_w_ ∼ 6000 g/mol) were dried and activated at 200 °C under an ultrahigh vacuum for 3 h. The desired wt % of Cu(I)-COF@PDMS-MA was then added to the liquid PDMS in a He glovebox and stirred until suspended. The porous liquid was again dried for 1 h at 200 °C under an ultrahigh vacuum prior to further study.

### 2.3 Instrumentation

#### 2.3.1 NMR Spectroscopy

^1^H NMR (400 MHz) spectra were recorded at rt on a Bruker Avance III HD NanoBay NMR spectrometer, locked on the signal of CDCl_3_.

#### 2.3.2 Powder X-ray Diffraction (XRD)

Powder XRD measurements were performed on a PANalytical PW3040 X-ray diffractometer using Cu Kα (λ = 1.54 Å) radiation. The scan rate was 2°/min with a current of 40 mA and a voltage of 45 kV.

#### 2.3.3 Physisorption Measurements

Isotherms were collected on a Micromeritics ASAP 2020. Each sample was degassed at 200 °C prior to analysis and transferred to a physisorption instrument without air exposure. CO_2_ isotherms were collected at 0 °C with a 30 s equilibration time for each data point. A density-functional theory (DFT) slit-pore model was used to extract the pore size distributions.

#### 2.3.4 Differential Scanning Calorimetry

The glass-transition temperatures were obtained with a TA Instruments DSC 25 equipped with a Discovery liquid N_2_ pump, allowing a minimum sampling temperature of −150 °C. The system was calibrated at a given temperature ramp using an indium reference sample prior to sample measurements. The samples were heated at a 10 °C/min ramp rate with a 50 mL/min N_2_ flow through the cell and a 307 mL/min base purge.

#### 2.3.5 Polymer Molecular Weight Determination

Polymer samples were dissolved in HPLC-grade tetrahydrofuran (THF) (∼1 mg/mL), filtered through alumina to remove the Cu catalyst, and then filtered through a 0.45 μm PTFE filter. Size exclusion chromatography was then performed on a PL-Gel 300 × 7.5 mm (5 μm) mixed C column using an Agilent 1200 series autosampler, an inline degasser, and a diode array detector set to monitor the absorbance at 254 nm. The column and detector temperatures were 35 °C. HPLC-grade THF was used as the eluent (1 mL/min). Linear polystyrene standards were used for calibration.

#### 2.3.6 Scanning Transmission Electron Microscopy (TEM)

Scanning TEM images and the corresponding energy-dispersive X-ray spectroscopy (EDS) hypermaps were collected using an FEI Talos F200X operated at 200 kV. Samples were suspended in hexane and dropped onto a 300-mesh gold grid with lacey Formvar/carbon (Ted Pella, 060821). Elemental EDS maps were both collected (acquisition time 5 min) and processed by standard methods using Bruker ESPRIT software.

#### 2.3.7 UV–Vis Spectroscopy

Absorbance measurements were performed at rt on a Varian Cary 50 Scan UV–visible spectrophotometer. Samples were prepared in a PDMS solvent under a N_2_ atmosphere and sealed in a 1 cm quartz cuvette prior to analysis.

#### 2.3.8 Dynamic Light Scattering (DLS)

Particle size distributions were measured with a Malvern Panalytical Zetasizer Nano series instrument in a quartz cuvette. Measurements were performed in acetonitrile. Three consecutive measurements were performed to confirm that aggregation was not occurring on the time scale of the measurement.

#### 2.3.9 Diffuse Reflectance Infrared Fourier Transform Spectroscopy (DRIFTS)

DRIFTS measurements were performed in a Thermo Scientific Nicolet iS50 FT-IR spectrometer equipped with a Harrick Scientific Praying Mantis reaction chamber. Unless otherwise noted, the gas flow was set to 100 sccm. The samples were pretreated at 200 °C under an ultrahigh vacuum for 3 h. After cooling, the samples were sealed in an inert environment. The reaction chamber was purged with He at 100 sccm for ca. 10 min after loading the sample in an inert environment. A background spectrum was then collected. The Cu–COF@PDMS-MA, neat PDMS, and Cu–COF@PDMS-MA porous liquid were each exposed to a 10% CO (CO/He) mixture for 30 min (Cu–COF@PDMS-MA, neat PDMS) or 1 h (Cu–COF@PDMS-MA porous liquid) at rt. The samples were then purged with He for 40–60 min to remove any free CO before collecting the spectrum at a ramp rate of 5 °C/min.

#### 2.3.10 Temperature-Programmed Desorption (TPD)

TPD measurements were performed on a calibrated, custom-built system equipped with a Stanford Research Systems RGA 100, capable of measuring *m*/*z* = 1–100 amu. An *m*/*z* range of 1–50 amu was used for each experiment. Prior to analysis, the COF colloids (stored in dichloromethane) were isolated by removing the solvent on a rotary evaporator, dried on a Schlenk line vacuum for 48 h at 90 °C, and further activated for 2 h at 200 °C under an ultrahigh vacuum. Analysis was performed on 2–5 mg of active COF material. The samples were dosed with 1.3 bar H_2_ for 10 min at rt (porous liquids were stirred during dosing), followed by a liquid N_2_ quench and evacuation of the head space until the H_2_ signal reached a baseline pressure of 10^–8^ Torr. A type K thermocouple was employed to monitor the temperature, and the samples were heated at 15 °C/min unless otherwise noted. Experimental parameters were controlled via a LabView interface that is connected to the RGA, heating system, and pressure gauges. The output signal from the mass spectrometer was divided by the total sample mass to get a normalized signal.

## 3 Results and Discussion

### 3.1 Synthesis and Stabilization of COF Colloids

**Scheme 1:**
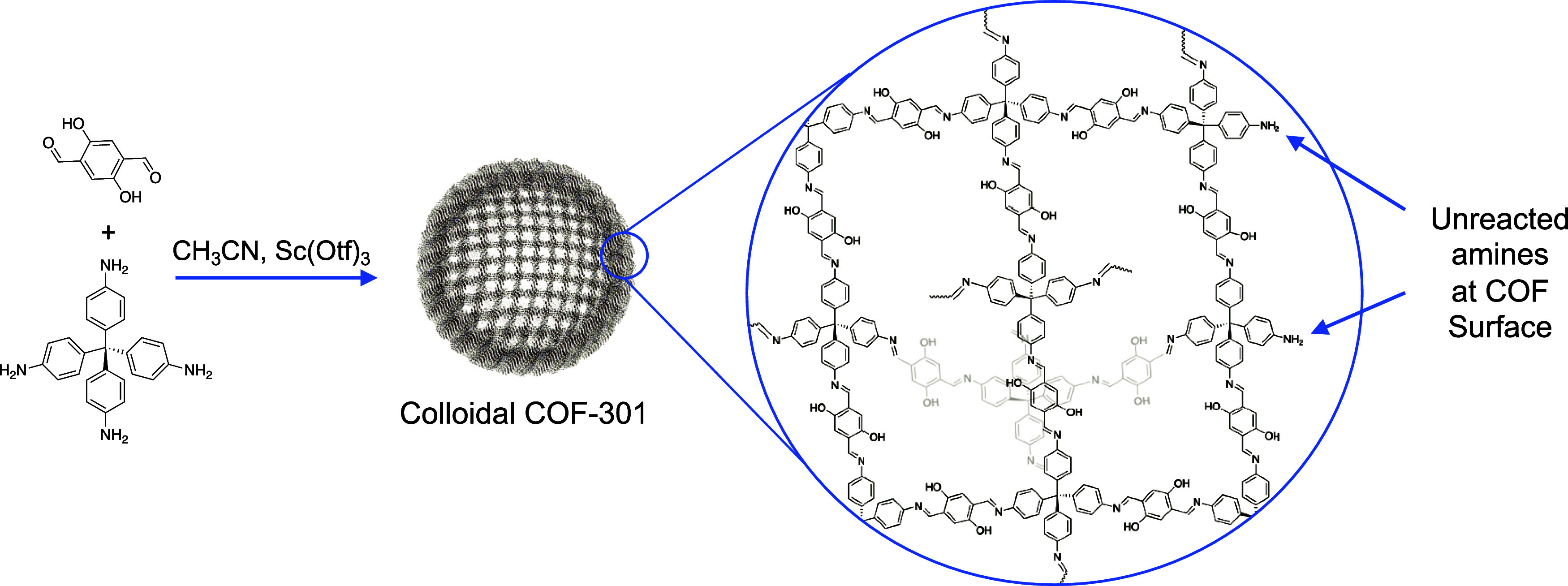
Synthesis of Colloidal COF-301

With the goal of synthesizing stable COF colloids containing open Cu(I) sites that could be employed for higher-temperature gas storage and transport in porous liquids, we focused our efforts on COF-301. In the context of any potential continuous flow applications, maintaining the long-term stability of a PL suspension is important. Aggregation or flocculation of the COF would result in an undesirable reactor fouling. Bulk COF-301 particles do not suspend well in our liquid polymer media; thus, in an attempt to enable the long-term stability of these suspensions, we focused our efforts on synthesizing and preserving the COF material in a colloidal state. The 3D imine-based COF-301 was previously synthesized from tetrakis(4-aminophenyl)methane and 2,5-dihydroxy terephthalaldehyde and has been identified as a promising candidate for H_2_ storage.^35^ We recently demonstrated that atomic Cu(I) could be installed in the phenol–imine docking sites of this COF with a postsynthetic modification and activation procedure, after which stable complexes with H_2_, C_2_H_4_, and CO could be formed and detected near ambient temperature in bulk COF powder.^34^ In other recent works, we developed a procedure for tuning the particle size of certain 3D imine-based COFs using dilute nitrile-based solvents and optimized catalysts.^24^ The latter conditions were applied herein to synthesize the first known colloids of COF-301 (Scheme 1) and subsequent COF-301-based PLs.

Under the conditions outlined in the Experimental Section, COF-301 formed spherical particles with a median diameter of 400 nm, as determined by dynamic light scattering (DLS) (Figure S7) and observed with TEM imaging. The Brunauer–Emmett–Teller (BET) surface area was approximated as 400 m^2^/g (Figure S4), on par with colloidal COF-300 literature analogues,^24^ and the pore size distribution of the COF-301 colloids revealed a strong monodisperse peak near 6 Å (Figure S5). Optimization of COF surface areas and crystallinity is typically achieved by modifying a slew of synthetic conditions, including the polarity of the solvent, the reagent and catalyst concentration, the moisture content, the reaction time, and temperature.^36,37^ Our group^34^ and others^38,39^ have reported synthetic procedures that enable higher-surface-area, crystalline COF-301. However, powder XRD measurements on this colloidal COF-301 material suggest that it is essentially amorphous in nature, characterized by broad and weak diffraction peaks (Figure S3). Given that these COFs remain colloidal only in nitrile-based solvents for about 72 h in the presence of a catalyst, additional optimization was not possible beyond tuning concentration and temperature. Nevertheless, crystallinity is not a fundamental prerequisite for efficient gas storage and separation in these materials.

After their synthesis, the colloids were purified by removing the unreacted monomer and acid catalyst through centrifugation (14,000 rpm, 15 min), decanting the supernatant, and immediately resuspending the colloids in fresh acetonitrile. The COF-301 colloids remained in suspension with minimal flocculation in acetonitrile for several months. Any particles that settled out of the solution could be readily resuspended with agitation. However, if dried or placed in a different (co)solvent suspension, irreversible aggregation occurred rapidly. The standard procedure for postsynthetic loading of COF-301 with metal salts (e.g., stirring the COF in a Cu(II) formate solution^33^) also induced irreversible aggregation of the colloids, presenting a challenge on how to incorporate Cu binding sites into these materials while keeping them colloidal.

We recently developed a surface functionalization technique for stabilizing COF colloids that involved tethering a bulky imidazolium salt to unreacted amines on the colloid surface; the strategy proved effective at stabilizing COF colloids in a variety of organic solvents and ionic liquids.^24^ We applied this strategy to the COF-301 colloids, whereby it was possible to load these stabilized colloids with Cu(II) formate. The colloids turned from yellow-orange to red-brown upon loading with Cu salt. The surface-functionalized colloids were thoroughly dried under a vacuum and resuspended in common organic solvents. However, upon activating the COF and converting the Cu(II) formate to open Cu(I) sites in these materials by heating for several hours at 200 °C (per recent literature procedures for analogous compounds),^33,34^ the colloids aggregated irreversibly. Although stable at 100 °C, it appears that the tethered imidazolium salt is not sufficiently robust at temperatures required to generate open Cu(I) sites with this technique.

We then implemented a more robust surface functionalization strategy to realize Cu(I)-loaded COF colloids suitable for PL applications. A variety of surface functionalization techniques have been employed in the MOF literature for improving colloid stability or dispersibility,^40−43^ tuning interfacial properties,^44−47^ and influencing gas permeation^48,49^ or drug release.^50,51^ However, these techniques are not always compatible with or generalized to the specific surface functionalities of COFs. A strategy to functionalize and stabilize COF colloids with a robust polymer coating would not only enable the development of stable Cu(I)-containing PLs but such a technique could also facilitate the advancement of other highly tunable COF@polymer hybrid materials.

**Scheme 2:**
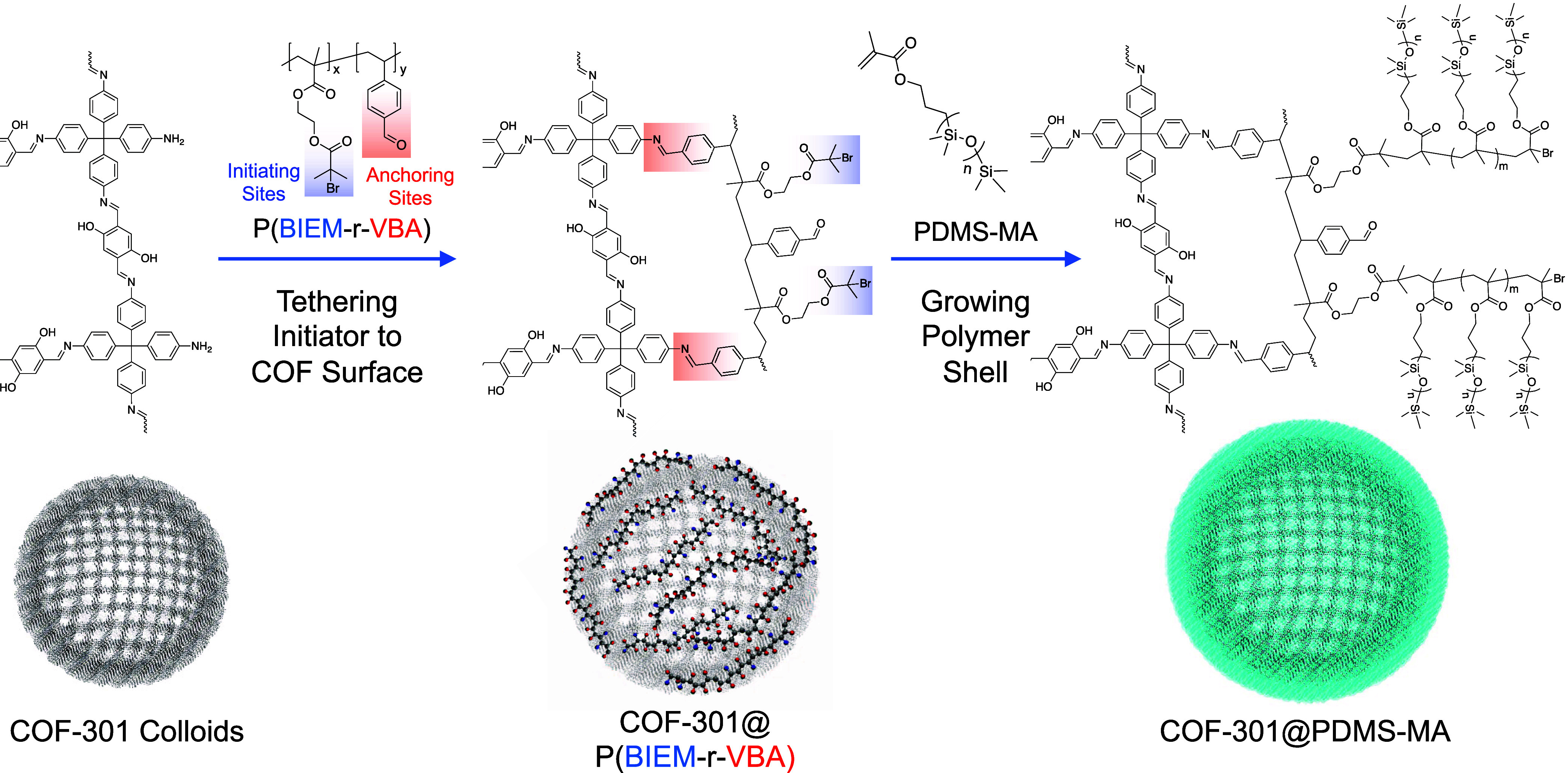
Dual Functional Copolymer Was Synthesized with Benzaldehyde “Anchoring” Sites and 2-Bromoisobutyryloxy “Initiating” Sites After tethering This copolymer to COF colloids through a surface condensation reaction, a poly(dimethylsiloxane)-methacrylate (PDMS-MA) membrane is grown around the COF using atom transfer radical polymerization (ATRP).

One literature technique that was potentially attractive for our system utilized a dual-functional copolymer to coat MOF colloids and generate MOF-based PLs.^42,43^ In this process, carboxylic acid groups in the copolymer tethered it to the surface of the MOF, while Br-functionalities in the copolymer could be used to initiate additional polymerization growth from and around the surface of the MOF. Here, we were able to tailor the synthesis of a new copolymeric initiator specifically for application with imine-based COFs; using radical addition–fragmentation chain-transfer (RAFT) polymerization,^52,53^ we synthesized a copolymer of 2-(2-bromoisobutyryloxy)ethyl methacrylate (BIEM) and 4-vinyl benzaldehyde (VBA) (see the Experimental Section for details). The aldehyde groups in this random copolymer P(BIEM-*r*-VBA) (Scheme 2) could then react with surface amines of COF-301 colloids, effectively acting as a multidentate binding agent that formed a thin polymeric coating on the surface of the colloids. Once this thin coating was in place, we discovered that the colloids were now stable in solvents other than acetonitrile, at least for several days. This allowed the colloids to be suspended in a variety of additional solvents and monomers, from which thicker, more robust polymer coatings could be grown via atom transfer radical polymerization (ATRP)^54−56^ using the Br-sites in the P(BIEM-*r*-VBA) coating as initiators.

In the following sections, we outline the conditions for (1) growing polymeric coatings based on poly(dimethylsiloxane)-methacrylate (PDMS-MA) of controlled thicknesses around COF colloids; (2) loading these coated colloids with Cu and characterizing the activated materials; and (3) studying and characterizing the porous liquid character of these materials for H_2_ applications.

### 3.2 Model Polymerizations

Poly(dimethylsiloxane) (PDMS) is an attractive material for PL gas storage and transport for several reasons: PDMS is inexpensive, nontoxic, and has a low vapor pressure, high thermal stability, and good H_2_ solubility. The fluidity of PDMS down to −40 °C (Figure S8) also makes the solvent promising for low-viscosity PL applications. While linear PDMS cannot be easily grown as a robust coating around framework-based colloids using conventional polymerization techniques, in principle, any vinyl monomer compatible with ATRP could be employed for the polymeric coating in this material. Thus, we chose PDMS-methacrylate (PDMS-MA) as a proxy for PDMS from which to synthesize the coating. The coated material could then be suspended in bulk PDMS to generate a fluid PL. However, these so-called “bottlebrush” polymers comprised of densely grafted side chains can be challenging to synthesize;^57^ control over polymerization chain length and dispersity is highly dependent on polymerization conditions (i.e., solvent, catalyst, temperature, and monomer concentration) and must be optimized for a given system. Here, we developed conditions that allowed us to control bottlebrush PDMS-MA synthesis while simultaneously keeping the COF-301 colloids suspended and well-dispersed.

An interesting feature of PDMS-MA brushes is their tendency to depolymerize under certain conditions, which is caused by a significant bond strain in these intrinsically bulky materials; significant work has been implemented to understand this phenomenon and the equilibrium between polymerization and depolymerization in the radical polymerization of these brush materials.^58,59^ Solvent selection, reaction temperature, and initial monomer concentration are all instrumental in influencing this equilibrium and promoting high yields and controlled molecular weights. High monomer concentrations drive polymerization, while good solvation of the polymer along with subsequent brush swelling and bond strain drive depolymerization. At some point during a reaction, the monomer concentration drops low enough to where polymerization and depolymerization are effectively in equilibrium, and chain growth halts.^60^

**Figure 1:**
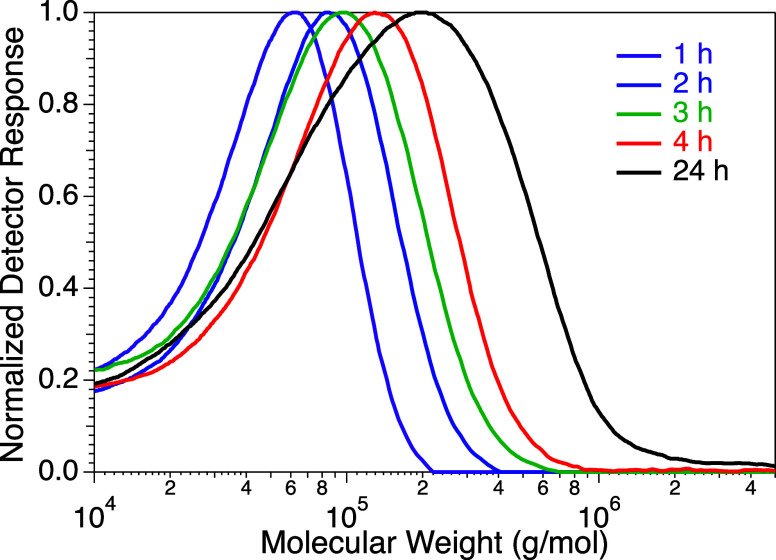
Gel permeation chromatography (GPC) traces illustrating PDMS-MA brush growth with time. Conditions: 200:1:2:2 PDMS-MA:EBriB:CuCl:Me_6_TREN, rt,10/90 mixture of acetonitrile/dioxane.

We first conducted a model ATRP reaction of PDMS-MA in acetonitrile, as this solvent would ultimately be optimal for keeping our colloids dispersed when later conducting surface-initiated ATRP. However, while the polymerization proceeded, the poor solubility/immiscibility of the PDMS-MA brushes in acetonitrile led to poorly controlled chain growth with large batch-to-batch variability. In pure dioxane, the brushes were well solvated, but both the ATRP catalyst and the colloids were not, leading again to uncontrolled polymer growth. We ultimately discovered a simple 90:10 mixture of dioxane:acetonitrile to be the best compromise for catalyst solubility, brush solubility, and colloid dispersion. By employing CuCl/Me_6_TREN as the catalyst at rt, per recent optimization studies conducted for these brushes in the literature,^59^ systematic chain growth between 20 and 35 kg/mol could be achieved using monomer:initiator ratios of 200:1 (Figure 1), with an ∼70% conversion reached in 24 h. By decreasing that ratio to 20:1, molecular weights below 10 kg/mol were achieved (Figure S10). Note that the estimated number average molecular weight was consistently lower than theoretical values, which is typical when estimating brush molecular weights using linear polystyrene standards for calibration.^61^ Regardless, these experiments provided us with conditions for later tuning the coating thickness on our colloids.

### 3.3 COF-301@PDMS-MA and Cu Loading

**Figure 2:**
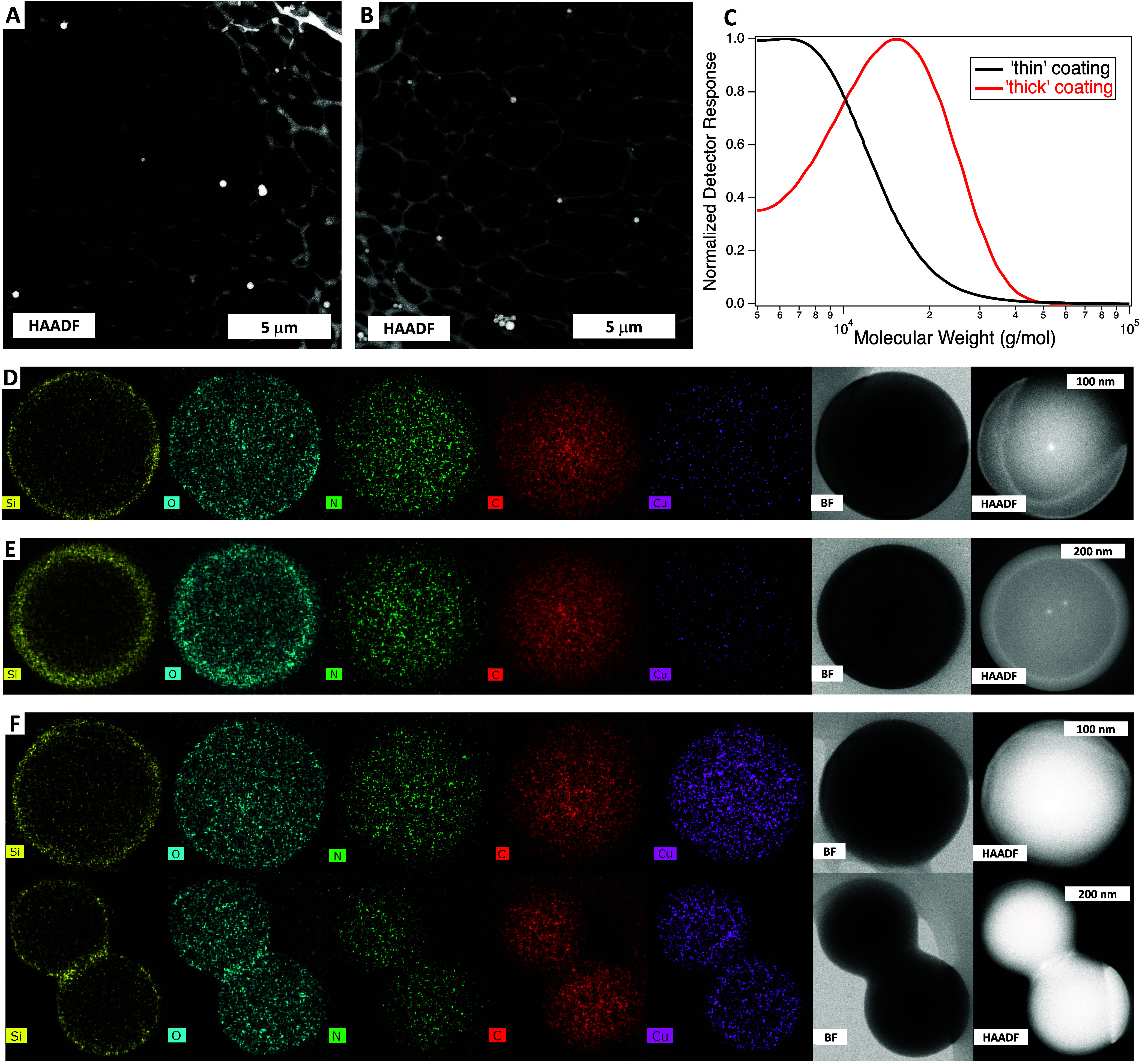
HAADF/scanning transmission electron microscopy (STEM) images showing the dispersion of COF-301@PDMS-MA particles following (A) their initial synthesis and (B) after the addition of Cu, several purification cycles, drying, and resuspension. (C) A sacrificial initiator was used in the synthesis of COF-301@“thin”PDMS-MA and COF-301@”thick”PDMS-MA; black and red size exclusion chromatography traces for samples taken from the thin and thick coating reactions, respectively. EDS mapping for Si (yellow), O (blue), N (green), C (red), and Cu (purple) for COF-301@PDMS-MA with (D) thin coating, (E) thick coating, and (F) thin coating following the addition of Cu(II)formate. N.B., different length scale bars in panels (D–F).

A PDMS-MA coating was grown from the surface of COF-301 colloids using the optimized procedure mentioned in the previous section and detailed in the Experimental Section. High-angle annular dark-field (HAADF) imaging, shown in Figure 2A, illustrates how individual particles of COF-301@PDMS-MA can be deposited from dichloromethane following the coating procedure. Notably, many of the particles remain individually dispersed following loading with Cu(II)formate (*vide infra*), purification *via* centrifugation, drying, resuspension in, and finally deposition from dichloromethane (Figure 2B); without the protective coating, this cannot be achieved. Additionally, in the “thick” coating procedure, we noted an average particle size increase from roughly 400 nm to approximately 460 nm postcoating, as determined via dynamic light scattering (DLS) (Figure S7). TEM analysis unveiled that the coating thickness falls within the range of 30–40 nm, generally consistent with the DLS data. However, the slight discrepancy may suggest that a small fraction of the particles experience permanent aggregation, which we also observe in the TEM images (e.g., Figure 2F).

The uniformity of the coating can be observed with scanning transmission electron microscopy (STEM) images of individual particles; furthermore, energy-dispersive X-ray spectroscopy (EDS) mapping was employed to clearly distinguish the outer coating from the inner COF core, as Si atoms were uniquely present in the coating and N atoms in the COF (Figure 2D–F). Trace Cu could also be observed in the coated COF (Figure 2D,E), attributed to the residual ATRP Cu catalyst. Variation of the monomer-to-initiator ratio during the polymerization afforded control over the coating thickness; thin coatings (Figure 2D, estimated by STEM to be ∼5 nm) were grown using a 20:1 monomer:initiator ratio, while thick coatings (Figure 2E, ∼ 30 nm) were achieved with a 200:1 ratio. An untethered “sacrificial” initiator was also injected during these surface-initiated polymerizations, as it has been demonstrated that tethered and untethered chains grow at approximately the same rate during living radical polymerizations.^62^ Thus, the molecular weights of the sacrificial chains measured using size exclusion chromatography (Figure 2C) by sampling the thin and thick reactions (*M*_n_ ∼ 5 and 15 kg/mol, respectively) can be considered a good estimate of the molecular weights of the individual chains tethered to the surface of the COF in Figure 2D,E. Elemental analysis suggested that the thick PDMS-MA shell constituted 25% of the overall sample mass, with the thin shell being 10% of the sample mass.

**Figure 3:**
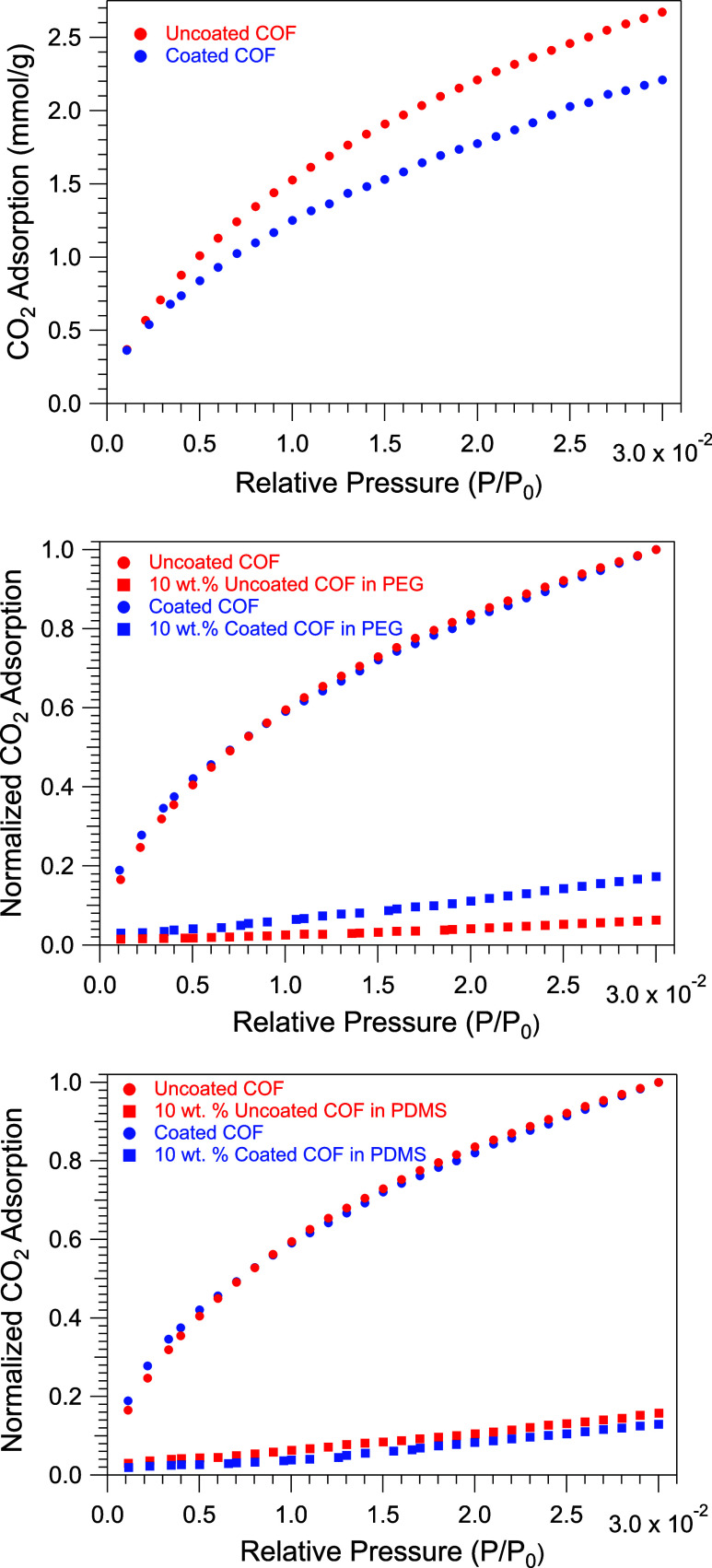
(Top) CO_2_ isotherms collected at 0 °C on the uncoated COF-301 (red) and COF-301@PDMS-MA-thick (blue), normalized to the total mass of the sample. (Middle and bottom) CO_2_ adsorption data at 0 °C depicted with a normalization factor applying setting adsorption to 1.0 at *P*/*P*_0_ = 0.03. To enable an effective comparison of the relative porosities retained in different porous liquid samples, the adsorption data for coated and uncoated 10 wt % PL were then plotted using the same normalization factor employed for either the coated or uncoated COF powders, respectively. CO_2_ adsorption for PEG-200-based porous liquids is illustrated in the middle graph, and PDMS-6000-based porous liquids are depicted in the bottom graph.

A Brunauer–Emmett–Teller (BET) surface area analysis using a N_2_ isotherm was not useful once the coating was applied, as the coating effectively acts as a glassy encapsulant to exclude N_2_ adsorption at cryogenic temperatures (*vide infra*). However, at 0 °C, where the coating is rubbery, the CO_2_ adsorption readily occurred. By comparing the quantity of CO_2_ adsorbed at any given relative pressure *P*/*P*_0_ between 0.01 and 0.03 in the isotherm in Figure 3 (top), we estimate that CO_2_ adsorption into COF-301@thick PDMS-MA is at least 80% of that of the uncoated material. When the mass was normalized to account for the sample coating, the adsorption of the active COF material remained essentially identical before and after the coating procedure.

Compared to uncoated COF colloids that irreversibly aggregate, the COF-301@PDMS-MA particles readily resuspended in PDMS and other solvents after drying at 200 °C in a high vacuum (10^–7^ Torr). Once stabilized by the coating, the colloids could also be loaded with Cu(II)-formate to introduce Cu(I) binding sites for H_2_ and other gases capable of π-backbonding. Cu has previously been incorporated at the phenol–imine site in bulk COF-301 by stirring in a Cu(II)formate methanol solution.^34^ However, we found this procedure inadequate for introducing Cu into the coated COF, possibly due to the poor miscibility of methanol with the PDMS-MA coating. By simply changing the solvent to a 1:1 mixture of dichloromethane and methanol, which visibly swelled the coating, Cu(II) formate appeared to readily permeate the un-cross-linked PDMS-MA coating and was uptaken by the COF. Similar materials were made using the added cross-linker in the synthesis of the membrane (see TEM images in Figures S11 and S12). However, when the membrane was cross-linked, Cu(II)formate uptake took place slowly over ∼1 week vs. mere hours when not cross-linked. Cross-linking also appears to influence gas diffusion, *vide infra*.

The COF changed color from orange to red-brown upon the addition of Cu (Figure S13). Features in the Fourier-transform infrared spectroscopy (FT-IR) spectra that shifted upon Cu loading were consistent with binding at the phenol–imine site (e.g., a shift in the imine stretch from 1610 to 1590 cm^–1^ was observed; Figure S14).^34^ Inductively coupled plasma (ICP) analysis was employed to estimate the metal-loaded sample to be 7 wt % Cu, similar to the 10 wt % Cu that the uncoated COF-301 will uptake with this procedure.^34^

Following heating of the Cu(II)–COF-301@PDMS-MA at 200 °C (see the Experimental Section), the Cu-loaded COF turns black, indicative of the formation of the Cu(I) species.^33^ The formate anion reduces Cu(II) to Cu(I) at these temperatures, evolving the synthesis of CO_2_ and H_2_ in the process. We have previously confirmed the oxidation state of Cu in analogous COF-301 materials using X-ray photoelectron spectroscopy (XPS);^34^ however, we found that the coating makes these materials highly insulating, not conducive for accurate XPS measurements. Thus, while the color change is qualitatively consistent with what we expected, we further probed the Cu oxidation state in these materials by studying the CO binding.

### 3.4 Gas Sorption in Porous Liquids

#### 3.4.1 Confirming Permanent Liquid Porosity

As previously mentioned, the BET surface area analysis using N_2_ isotherms does not yield significant insights when assessing the porosity of coated or porous liquid materials. This limitation arises from the dual roles played by the coating and the liquid matrix, which can act as impermeable encapsulants at cryogenic temperatures, hindering N_2_ adsorption. Nevertheless, CO_2_ isotherms conducted at 0 °C offer a viable alternative.

Several porous liquids were prepared as 10 wt % suspensions by adding PDMS-coated or uncoated COF-301 to bulk polymer liquids. We investigated both bulk PDMS (6 kg/mol, PDMS-6000) and bulk poly(ethylene glycol) (200 g/mol, PEG-200). The selection of the latter was based on its well-documented ability to effectively permeate sub-5 Å pores.^63^ We aimed to investigate the effectiveness of our coating in excluding such a small polymeric molecule from COF-301. The samples were allowed to stir at rt for 3 days. CO_2_ adsorption isotherms were then recorded and are plotted in Figure 3 (middle and bottom). A description of how these data were normalized is recorded in the figure caption. The middle graph of Figure 3 compares isotherms for the COF powders to that of the PEG-based porous liquids. The adsorption profile of the 10 wt %-coated COF PL in PEG-200 suggests that the coated COF fully retains its porosity in this medium; the PL adsorbs 1/10th the CO_2_ as the coated COF powder, and adsorption by neat PEG-200 at these pressures is comparatively negligible (Figure S6). Conversely, the relative CO_2_ adsorption in the uncoated COF PL sample is significantly lower, measuring 1/3rd that of the coated PL. The latter result suggests that substantial infiltration of PEG-200 occurs into the pores of the uncoated COF.

Remarkably, both 10 wt % suspensions of COF-301 in PDMS-6000, whether coated or uncoated, exhibited similar CO_2_ uptake at 0 °C (see Figure 3, bottom). Both adsorption isotherms suggest that the COF porosity remains preserved within PDMS-6000. Furthermore, these isotherms remained virtually unchanged even after the samples were allowed to stir for 1 week at 90 °C. The compact pore size of 6 Å in this COF appears effective at preventing significant penetration of linear PDMS-6000 over the duration of this experiment. This observation aligns with findings in the literature, where it has been noted that a MOF featuring even larger 12 Å apertures can exclude PDMS-4000 for a period spanning several months.^42^ Although the CO_2_ uptake was remarkably similar for the coated COF and uncoated COF-based porous liquids, one advantage of coated COF was improved suspension stability. Although the coated COFs began to flocculate after a few days suspended in bulk PDMS, they readily redispersed with agitation. The uncoated materials rapidly and permanently aggregated in PDMS.

#### 3.4.2 Diffuse Reflectance Infrared Fourier Transform Spectroscopy (DRIFTS)

**Figure 4:**
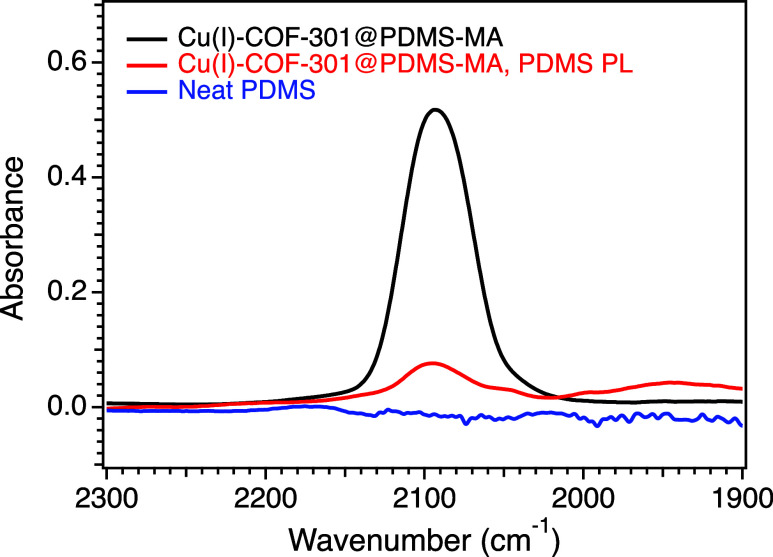
DRIFTS spectra of solid-state Cu(I)–COF-301@PDMS-MA (black), a porous liquid made from Cu(I)–COF-301@PDMS-MA suspended in liquid PDMS (10 wt % COF) (red), and neat PDMS (blue) after exposure to 10% CO in He at rt, followed by a 2 min He purge. The stretch observed at 2093 cm^–1^ is associated with CO bound to the Cu(I) site, red-shifted from the unbound CO centered near 2143 cm^–1^.

The presence of an available coordination site, namely, the Cu(I) moiety in Cu(I)–COF-301@PDMS-MA, was confirmed by dosing the system with carbon monoxide (CO) and studying its sorption using DRIFTS. A stretch associated with the CO–Cu(I) complex was observed at 2093 cm^–1^ in Figure 4 (black trace). This red shift from unbound CO (centered near 2143 cm^–1^) is consistent with previous reports of CO–Cu(I) complexes and indicates strong π-backbonding interactions (Figure S15).^34,64^ The stretch at 2093 cm^–1^ appeared immediately after exposure to 10% CO in He, and the CO remained bound to the Cu(I) site when the sample was purged at room temperature with He for 40 min. The sample was then heated at 10 °C/min to begin desorbing CO. The CO–Cu(I) stretch fully disappeared once the sample reached 84 °C (Figure S16).

A PL was then made by suspending activated Cu(I)–COF-301@PDMS-MA in liquid PDMS (dried at 200 °C), and the retention of the Cu(I) site activity in the PL was confirmed by the appearance of the same CO–Cu(I) stretch at 2093 cm^–1^ in DRIFTS (Figure 4, red trace). The neat PDMS spectrum confirmed that this stretch was CO bound to the Cu(I) site, rather than CO dissolved in the PDMS matrix (Figure S17). The kinetics of CO adsorption/desorption were clearly affected by the PDMS matrix, with the CO–Cu(I) stretch intensity slowly increasing during an hour of exposure to CO. Likewise, the CO–Cu(I) stretch never fully disappeared upon heating to 124 °C, at least not on the time scale of the experiment (Figure S18). The results indicate that the matrix has a strong influence on gas diffusion rates. This phenomenon was not unexpected but warranted further investigation.

We have previously been able to observe a broad, weak resonance near 3000 cm^–1^ associated with Cu(I)–H_2_ in DRIFT spectra;^33^ however, we were unable to observe such a resonance in these materials. Thus, we turn to another technique for evaluating the H_2_ sorption in these materials.

#### 3.4.3 Temperature-Programmed Desorption (TPD)

We and others have previously used TPD to evaluate H_2_ sorption in different Cu(I)-based MOFs^65^ and COFs,^33^ and we use it here to probe H_2_ diffusion in coated powder samples and in Cu(I)–COF-based PLs. Additionally, we probe diffusion above and below the glass-transition temperature (*T*_g_) of the polymer coating and liquid matrix. The *T*_g_ is a parameter of interest because gas diffusion through a polymer is typically associated with molecular motions that produce free volume and dynamic void spaces in the matrix.^66^ Above a polymer’s *T*_g_, substantial segmental motion typically results in higher gas diffusion coefficients by an order of magnitude or more. Sub-*T*_g_ chain end motions, such as those associated with the methyl groups in PDMS, are believed to be responsible for facilitating any gas diffusion that does occur below polymer *T*_g_.^67^

**Figure 5:**
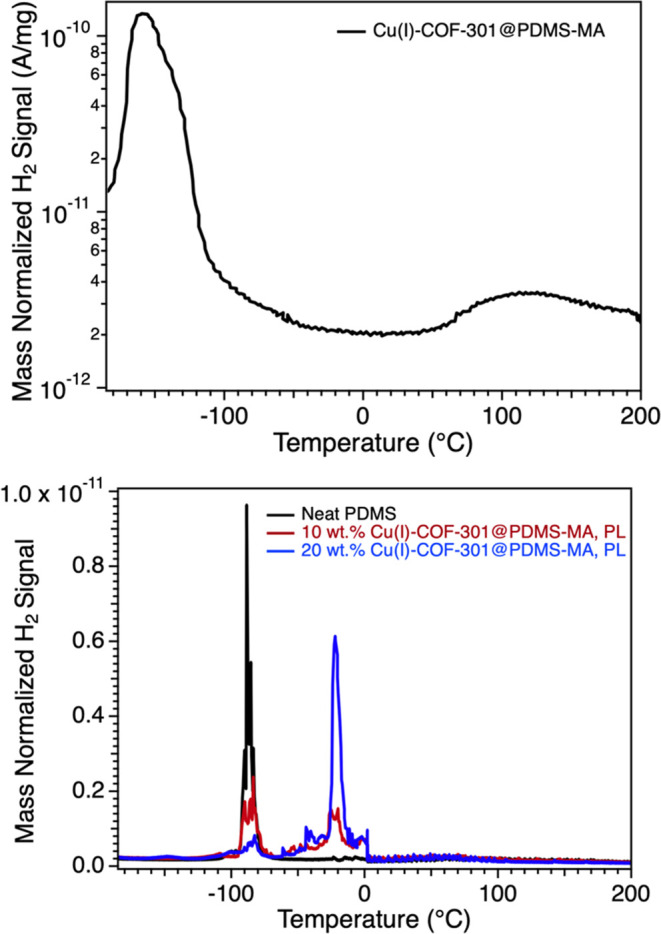
(Top) H_2_ TPD trace for Cu(I)–COF@PDMS-MA. Desorption at *T*_max_ = −150 °C is associated with H_2_ physisorbed to the parent COF and desorption at *T*_max_ = 115 °C is associated with H_2_ desorbing from the Cu(I) site. (Bottom) H_2_ TPD traces for neat PDMS (black) as well as porous liquids made by suspending 10 and 20 wt % of Cu(I)–COF-301@PDMS-MA in liquid PDMS. All traces are normalized to the total sample mass.

We first looked at H_2_ diffusion in the coated Cu(I)–COF-301 material. In these samples, the coating was not cross-linked, which facilitated better Cu uptake. In these experiments, the samples were first dosed with 1.3 bar H_2_ at rt for 10 min, after which they were quenched in liquid N_2_. The head space was evacuated until the H_2_ signal reached a baseline pressure of 10^–8^ Torr. The temperature was then ramped at 15 °C/min under high vacuum. Under these conditions, two distinct desorption peaks were observed for the coated Cu(I)–COF-301@PDMS-MA samples (top graph, Figure 5). The first peak occurs near −150 °C and is attributed to general H_2_ physisorption in the COF. Note that this value shifts slightly from a value of −170 °C observed for the uncoated material, *vide infra*. A second peak, albeit significantly weaker, is observed to peak near +115 °C. We attribute this to desorption from a Cu(I) site. That temperature and general behavior are consistent with desorption temperatures observed previously in Cu(I) 2D COFs^33^ and Cu(I) 3D COFs.^34^ Importantly, the high-temperature desorption peak is not observed if Cu–COF is not activated from Cu(II) to Cu(I).

Cu(I)–COF-based porous liquids were then probed with TPD. Two PL samples were prepared by suspending 10 and 20 wt % of Cu(I)–COF@PDMS-MA in liquid PDMS-6000, and H_2_ desorption was compared with the neat PDMS sample in the bottom graph of Figure 5. In the neat PDMS sample (black trace), the onset of H_2_ desorption begins near −100 °C and peaks near −80 °C, above its *T*_g_ of −127 °C (Figure S8). By comparison, a similar peak was observed near −80 °C in both PL samples, but in addition, a second H_2_ desorption event was observed to peak near −20 °C. We attribute this to the desorption of H_2_ from the COF within the PL. Regrettably, the high-temperature H_2_ peak associated with the Cu(I) site in these materials is not distinctly observed at a specific temperature, despite confirmation from DRIFTS that the Cu(I) site is available. We attribute this to the slow diffusion of H_2_ from the material across a broad temperature range that would be difficult to observe in this experiment. In any case, the clear desorption of H_2_ from the COF-based PL at refrigeration (as opposed to cryogenic) temperatures represents an exciting and noteworthy outcome.

**Figure 6:**
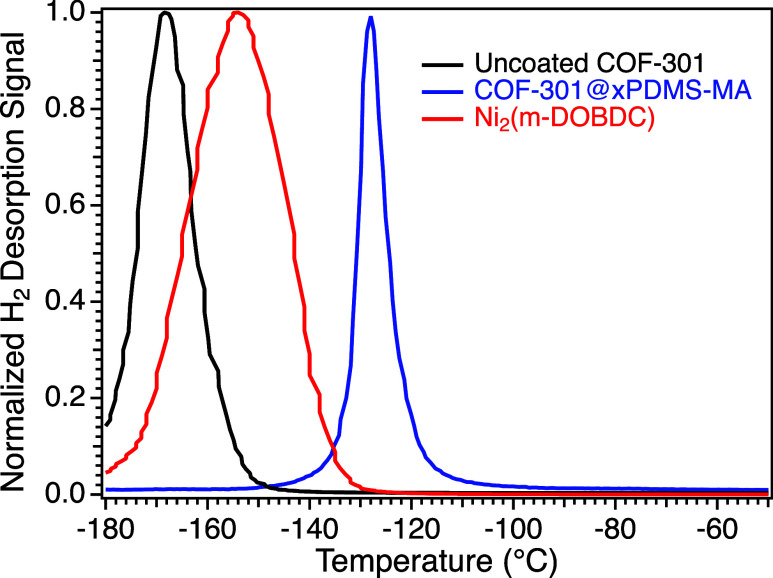
Normalized H_2_ desorption profiles for TPD measurements conducted under high vacuum illustrating H_2_ desorption from the uncoated colloidal COF-301 (black), COF-301@xPDMS-MA (blue), and the current state-of-the-art record-holding MOF for H_2_ storage near ambient temperatures, Ni_2_(m-DOBDC) (red). Samples were dosed at rt, cooled to 77 K, and then ramped at 15 °C/min under vacuum.

In Figure 6, we explore further how the coating can be used for tuning gas sorption kinetics, illustrating the desorption of H_2_ from uncoated colloidal COF-301 as compared to the thick coated COF-301@PDMS-MA. We also compare these desorption profiles to that of a sample of the current record-holding MOF in terms of capacity for near-ambient temperature H_2_ storage, nickel 4,6-dioxido-1,3-benzenedicarboxylate, or Ni_2_(*m*-DOBDC).^31^ Peak desorption (*T*_max_) occurred near −170 °C for uncoated COF and −154 °C for Ni_2_(*m*-DOBDC). Remarkably, peak desorption in the coated COF shifts to −128 °C, near the *T*_g_ of PDMS (Figure S8). On the time scale of the experiment, it appears that the PDMS-MA coating effectively confines H_2_ until the coating is heated close to its *T*_g_, at which point polymer segmental mobility would experience a noticeable uptick.

While the H_2_ capacity of these materials can in principle be measured with TPD, the technique can be unreliable when estimating the amount of weakly physisorbed H_2_ in a COF, as H_2_ will start to desorb from materials with a low binding enthalpy near 5 kJ/mol as soon as they are put under vacuum. According to “Chahine’s rule”^68^ and considering a surface area of merely 400 m^2^/g, it is expected that this COF material will capture less than 1 wt % of H_2_ under ideal conditions. Therefore, in terms of capacity, it would not be competitive with record-holding MOFs. Nevertheless, the shift in the desorption profile of the COF material depicted in Figure 6 upon coating the sorbent underscores a largely unexplored but potential benefit of these materials. We probe this phenomenon further with additional experiments in the Supporting Information (SI), illustrating how the coating thickness and the use of a cross-linking agent can influence both H_2_ adsorption and desorption behavior at temperatures above and below the *T*_g_ of the coatings. The results as a whole suggest that more efficient H_2_ storage might be achieved in sorbent materials with otherwise low binding enthalpies through the judicious choice of coating compositions and storage and delivery temperatures.

## 4 Conclusions

A well-controlled ATRP procedure was developed to grow bottlebrush PDMS-MA under conditions that were also compatible with keeping COF colloids well suspended. This procedure was applied to grow uniform PDMS-MA coatings of various thicknesses on the surface of COF-301 colloids. The PDMS-MA coatings stabilized the COF particles toward irreversible aggregation, enabling for the first time Cu loading into COF colloids and its efficient activation to Cu(I) at 200 °C. Stable Cu(I)-based PLs could then be implemented by suspending various analogues of the coated COF material in PDMS. CO_2_ isotherms confirmed that the coated COF retained its porosity when suspended in liquid polymers, while CO binding quantified by DRIFTS confirmed the nature of the Cu(I) site. DRIFTS was also used to study CO diffusion from the Cu(I) site in the PLs, while H_2_ sorption and diffusion in the materials were monitored with TPD. While observing H_2_ sorption specifically associated with the Cu(I) site in the PL proved challenging, TPD studies unveiled a substantial influence of the coatings and liquid matrix in these materials on general H_2_ diffusion. This observation highlights a largely unexplored yet potentially advantageous aspect of these materials in tuning gas storage and delivery temperatures. Taken together, the results illustrate how COF-based PLs with tunable properties hold promise for applications in efficient and customizable gas transport processes.

## Supporting Information Available

^1^H NMR spectra, XRD patterns, isotherms, DSC data, size exclusion chromatograms, TEM images, UV–vis spectra, DRIFTS spectra, and additional TPD traces (PDF)
