# Supplementary material for: Polymer-Coated Covalent Organic Frameworks as Porous Liquids for Gas Storage
Source: Chem Mater. 2024 Jan 19;36(3):1579–90. doi: 10.1021/acs.chemmater.3c02828 (PMC10870717; doi:10.1021/acs.chemmater.3c02828)
Supplement: Supplementary file 1 — cm3c02828_si_001.pdf [file cm3c02828_si_001.pdf]

Supporting Information for:

Polymer-Coated Covalent Organic Frameworks as Porous Liquids for Gas Storage

Rachel E. Mow,[a,c] *Glory A. Russell-Parks*[b,c] *Grace E. B. Redwine,*[b,c] *Brittney E. Petel,*[d] *Thomas Gennett,*[a,b,c *] *, Wade A. Braunecker* [b,c*]

[a] Materials Science Program, Colorado School of Mines, Golden, CO 80401, United States

[b] Department of Chemistry, Colorado School of Mines, 1012 14th Street, Golden, CO 80401, United States

[c] Chemistry and Nanoscience Center, National Renewable Energy Laboratory, 15013 Denver West Pkwy, Golden, CO 80401, United States

[d] Catalytic Carbon Transformation and Scale-Up Center, National Renewable Energy Laboratory, 15013 Denver West Pkwy, Golden, CO 80401, United States

Email: tgennett@mines.edu, Wade.Braunecker@nrel.gov

Table of Contents

I. Nuclear Magnetic Resonance…………. ……………………..……………………….…..S2
II. X-Ray Diffraction ……………………..……………..…………………………………..S3
III. Isotherms and Pore Size Distribution …………………….….…………………………S4
IV. Dynamic Light Scattering……..………….……………………………..……………..S5
V. Differential Scanning Calorimetry………..…………..………………………………..…S6
VI. Gel Permeation Chromatography ………..…………..……………………………....….S7
VII. Transmission Electron Microscopy ………………..………………………….………...S8
VIII. UV-Vis Absorbance ………………………….......…………………….………..…..…..S9
IX. Infrared Spectroscopy ………..…………..……….………………...…………..….S10
X. Temperature Programmed Desorption ………….………..……………………………...….S14

I. Nuclear Magnetic Resonance

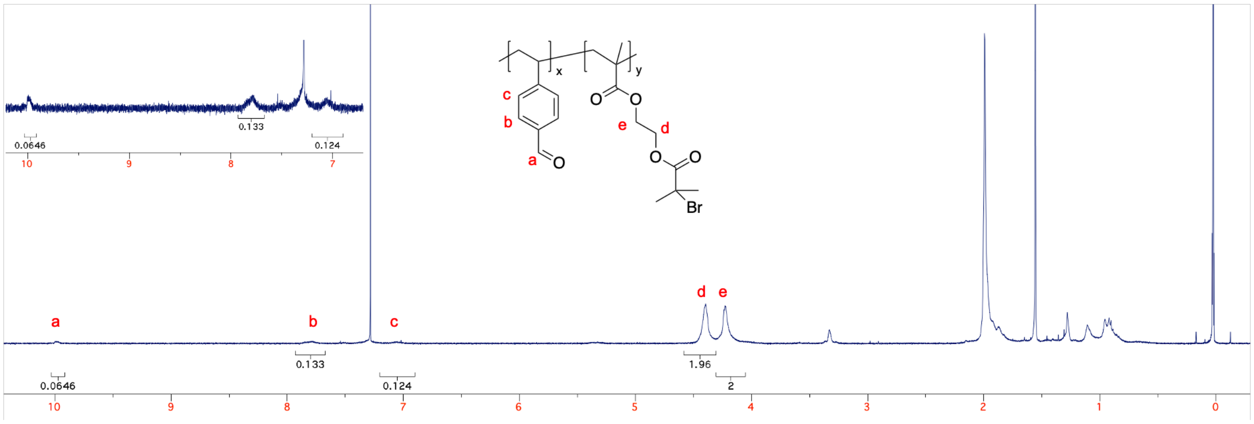

Figure S1. ^{1}H NMR spectra of P(BIEM-r-VBA), 400 MHz, $CDCl_3$. Integration indicates a 6:94 ratio of aldehyde tethering sites to Br-containing initiating sites.

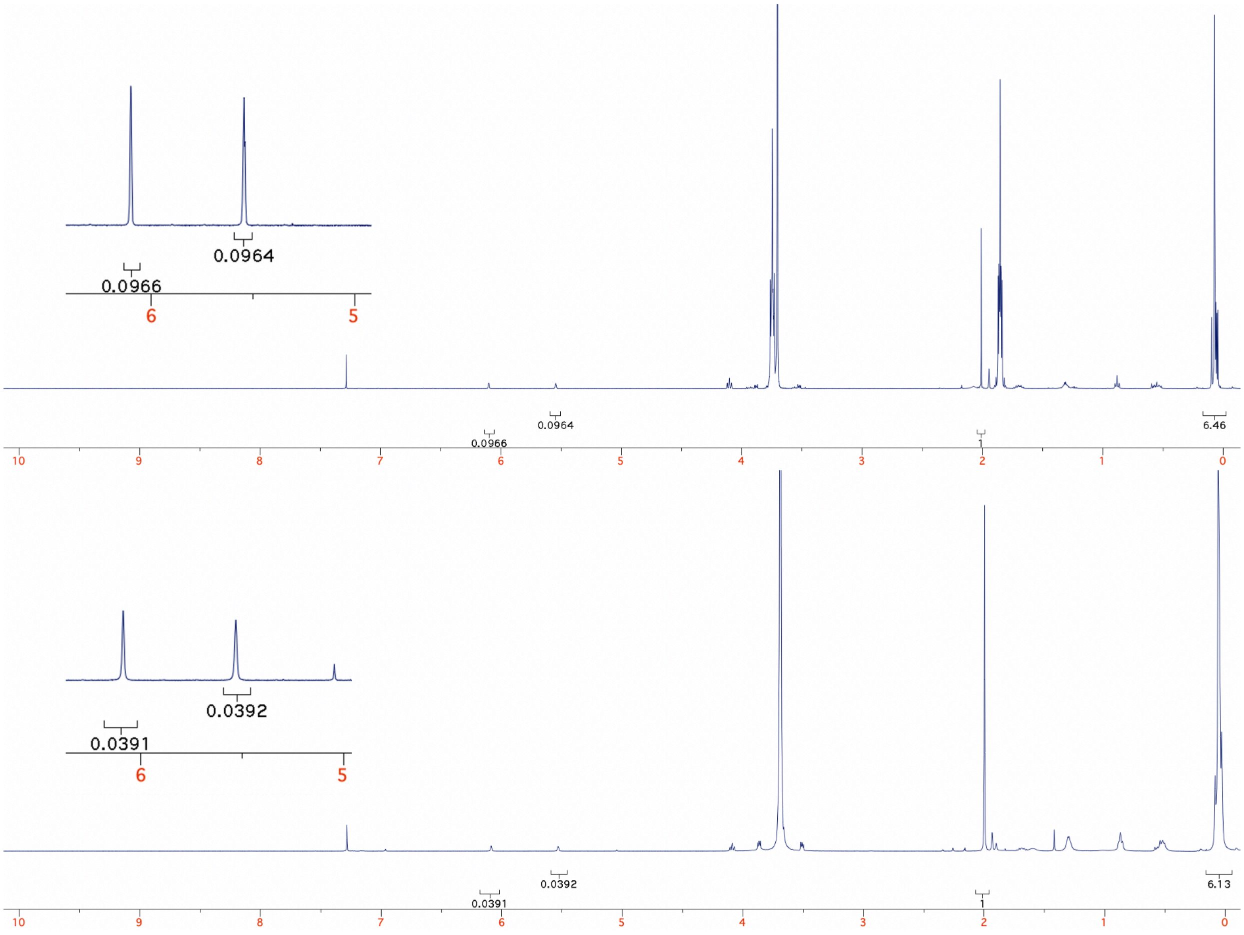

Figure S2. Representative ^{1}H NMR spectra employed for monitoring reaction conversion in the ATRP of brush PDMS-MA (200:1:2:2 PDMS-MA:EBriB:CuCl:Me_6TREN, 10/90 mixture of acetonitrile/dioxane, r.t.). Top: ‘T_0’ spectrum. Bottom: Spectrum taken from aliquot after 24 h.

II. X-Ray Diffraction (XRD)

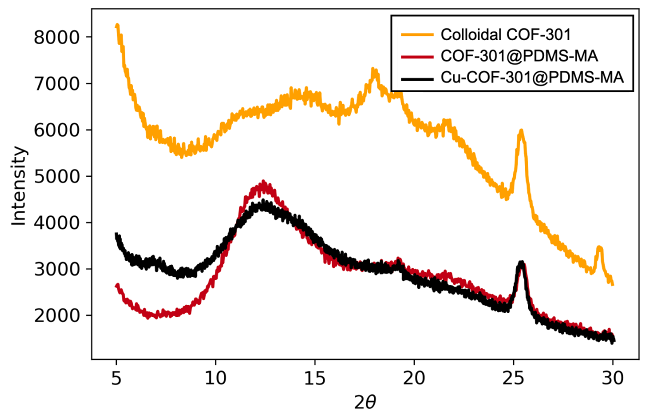

Figure S3. Powder XRD patterns of colloidal COF-301 (yellow), coated COF-301@PDMS-MA (red), and Cu(II) loaded COF-301@PDMS-MA (black).

III. Isotherms and Pore Size Distribution

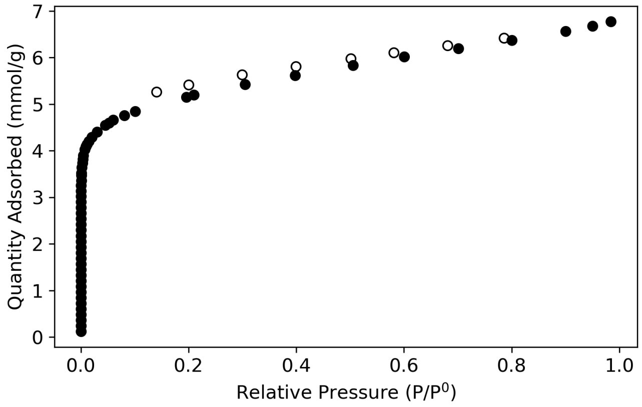

Figure S4. N_2 isotherm of the uncoated COF-301 colloids. BET analysis suggests 400 m^2/g surface area.

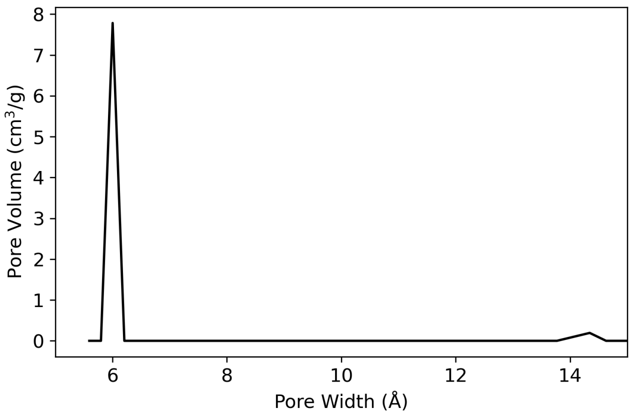

Figure S5. Pore size distribution of the uncoated COF-301 colloids from DFT slit-pore analysis of BET data.

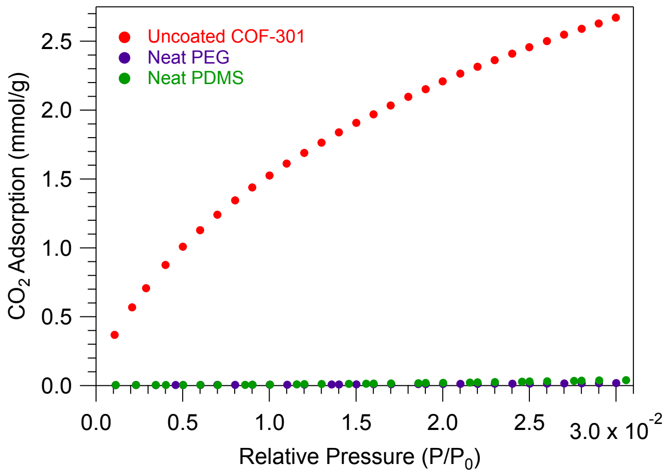

Figure S6. CO_2 isotherms collected at 0 °C on uncoated COF-301 (red), neat PEG (purple), and neat PDMS (green).

IV. Dynamic Light Scattering.

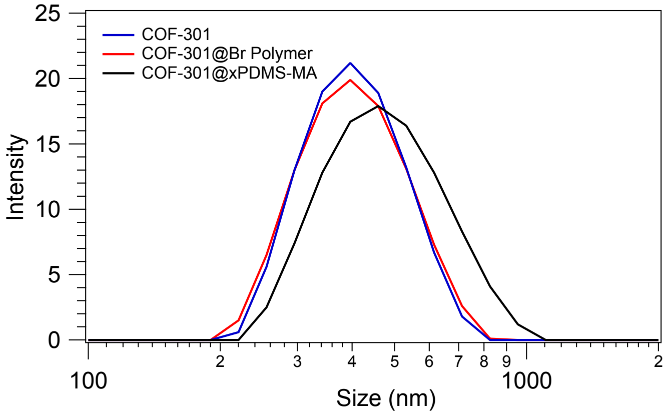

Figure S7. Dynamic light scattering data for colloidal COF-301 (blue), COF coated with Br-initiator (red), and COF-301@xPDMS-MA (black).

V. Differential Scanning Calorimetry

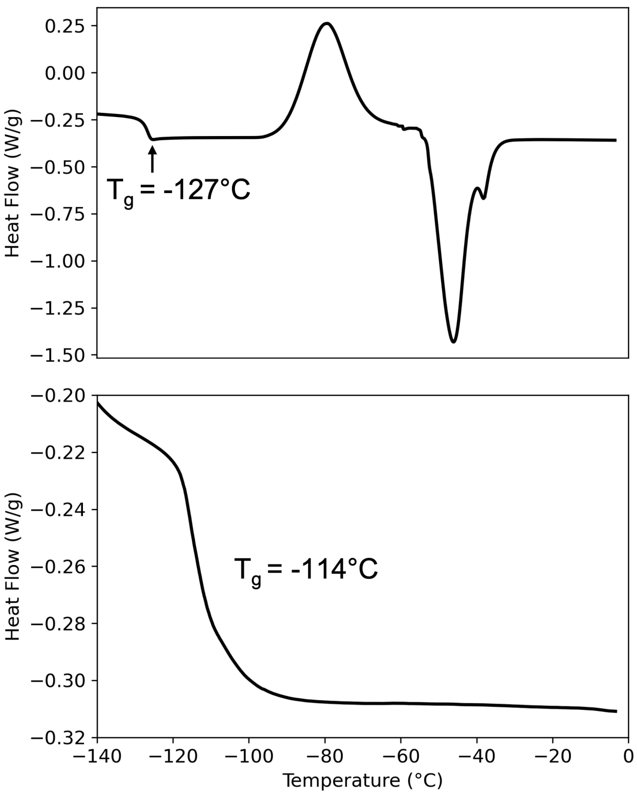

Figure S8. DSC traces for neat PDMS (top) and PDMS-MA brushes containing 10 wt.% crosslinker (bottom). Samples were flashed cooled, and data was collected during the heating cycle at a 10 °C/min ramp rate.

VI. Gel Permeation Chromatography

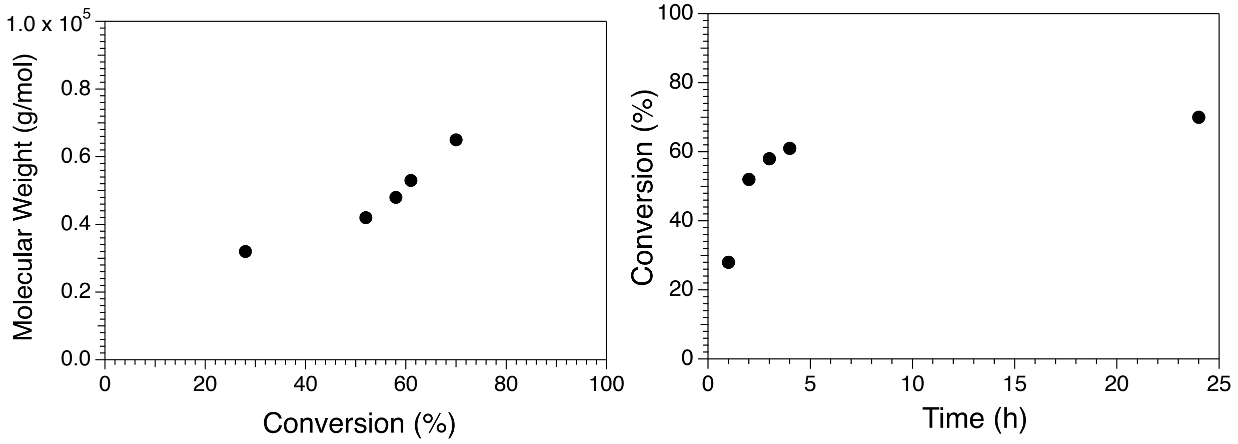

Figure S9. Molecular weight as a function of conversion (left), and conversion as a function of time (right). Conditions: 200:1:2:2 PDMS-MA:EBriB:CuCl:Me_6TREN, r.t.,10/90 mixture of acetonitrile/dioxane.

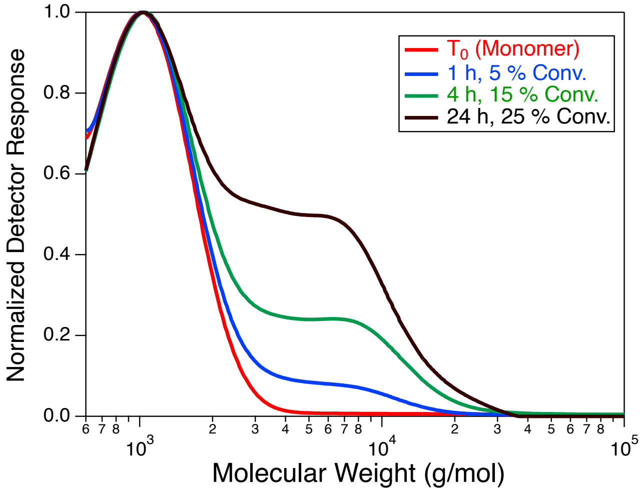

Figure S10. PDMS-MA molecular weight as a function of time and conversion. 20:1:2:2 PDMS-MA:EBriB:CuCl:Me_6TREN, r.t., 10/90 mixture of acetonitrile/dioxane. Conversion estimated from NMR.

VII. Transmission Electron Microscopy

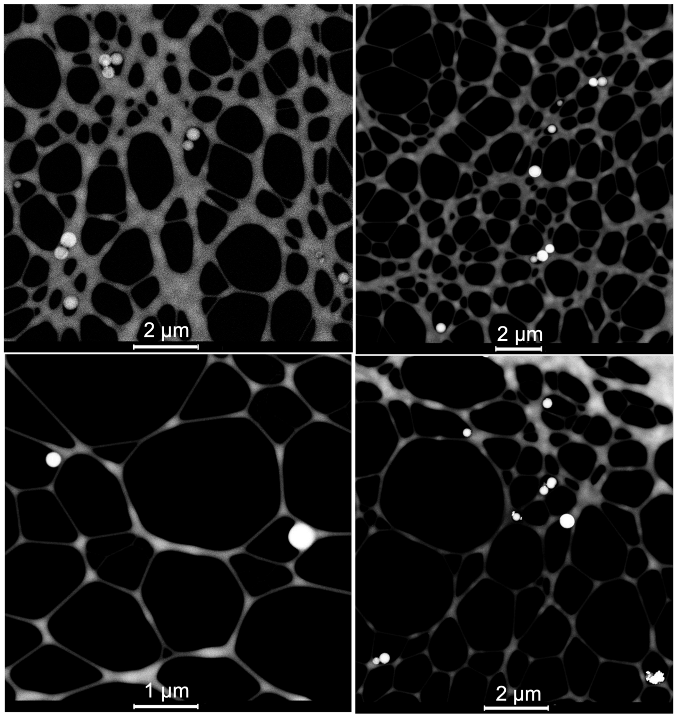

Figure S11. HAADF/STEM images of crosslinked COF-301@xPDMS-MA (top row) and crosslinked Cu-COF-301@xPDMS-MA, after loading with Cu(II)formate (bottom row). Synthesis conditions = 20:2:1:1 ratio of PDMS-MA:crosslinker:CuCl:Me_6TREN, r.t., 10/90 mixture of acetonitrile/dioxane.

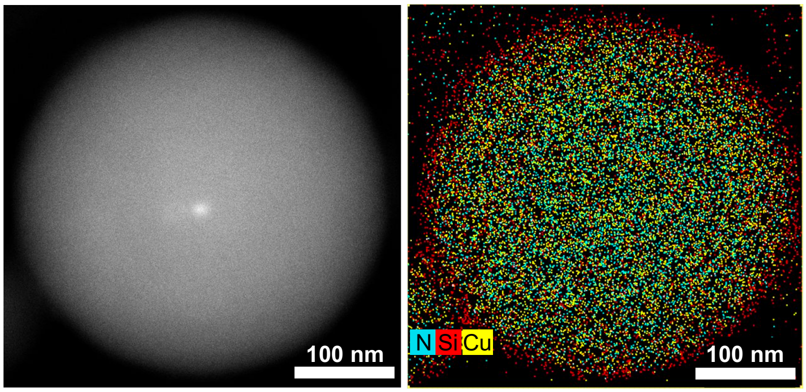

Figure S12. HAADF/STEM images and EDS mapping for Si (red), N (blue), and Cu (yellow) for crosslinked COF-301@xPDMS-MA with a 'thin' coating after addition of Cu(II)formate. Synthesis conditions = 20:2:1:1 ratio of PDMS-MA:crosslinker:CuCl:Me_6TREN, r.t., 10/90 mixture of acetonitrile/dioxane.

VIII. UV-Vis Absorbance

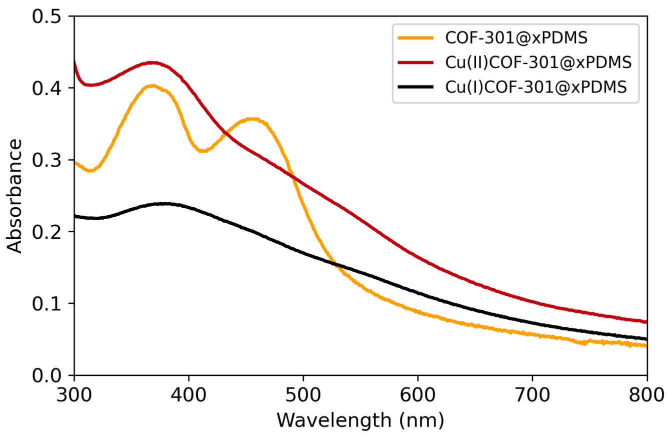

Figure S13. UV-Vis spectra of crosslinked COF-301@xPDMS-MA samples suspended in bulk PDMS at room temperature. COF sample prior to loading with Cu (orange), after loading with Cu(II) formate (red), and after activation to Cu(I) (black).

IX. Infrared Spectroscopy

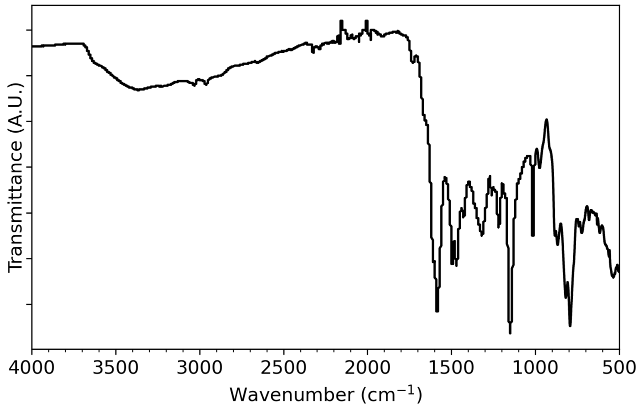

Figure S14. ATR spectra of Cu(II)-COF-301@PDMS-MA.

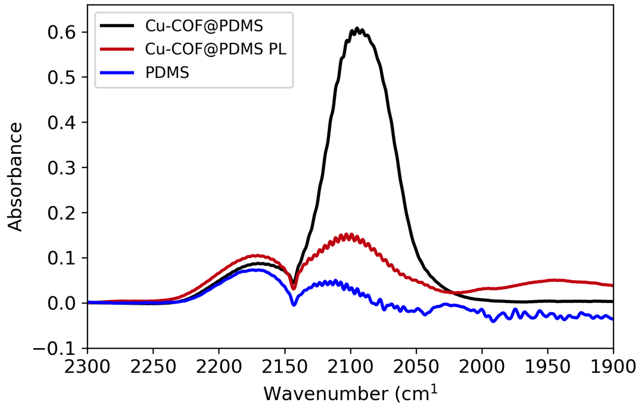

Figure S15. DRIFTS spectra of CO dosed into solid state Cu(I)-COF@PDMS-MA (black), CO in a porous liquid made from 10 wt.% Cu(I)-COF@PDMS-MA suspended in bulk PDMS (red), and CO in neat PDMS (blue).

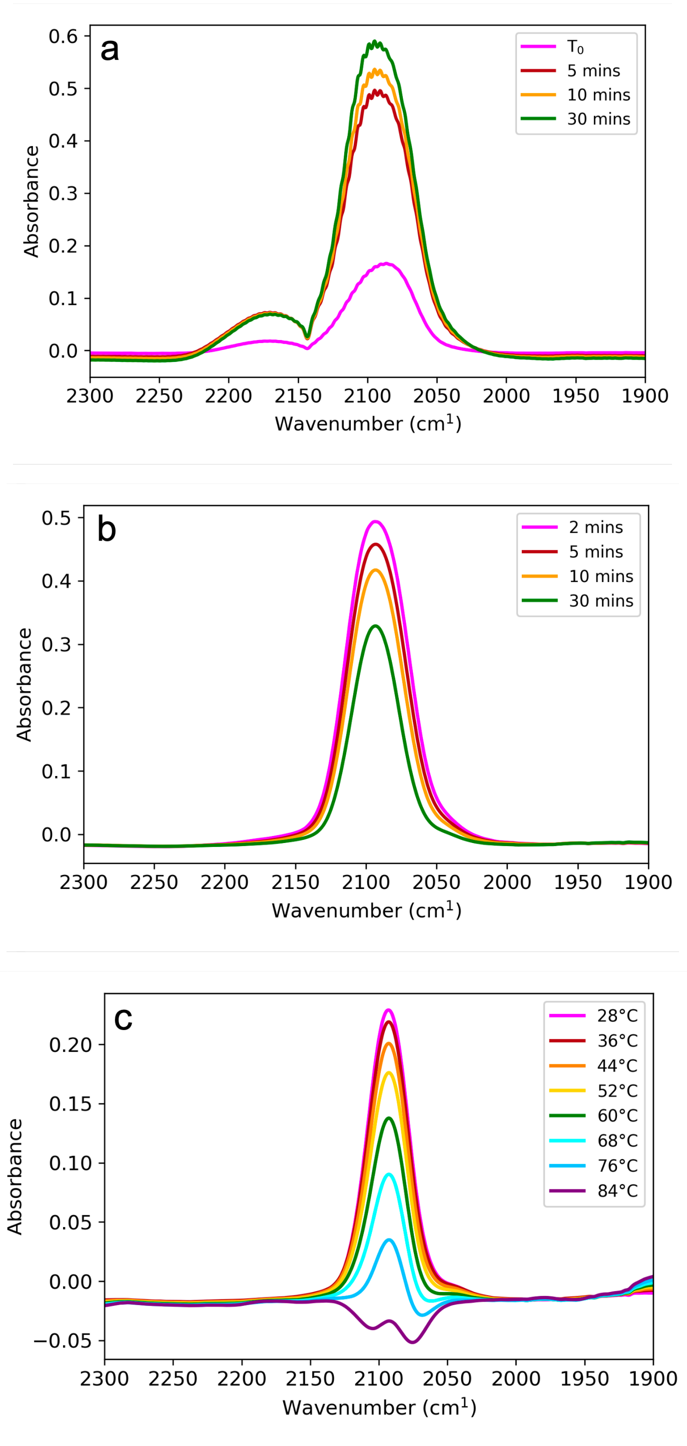

Figure S16. DRIFTS spectra of solid state Cu(I)-COF@PDMS-MA while a) flowing 100 sccm 10% CO in He, b) 100 sccm pure He following the CO dose, and c) heating at 10 °C/min under a He flow to follow CO desorption. Note, the background spectrum was collected at r.t., causing some baseline drift with heating.

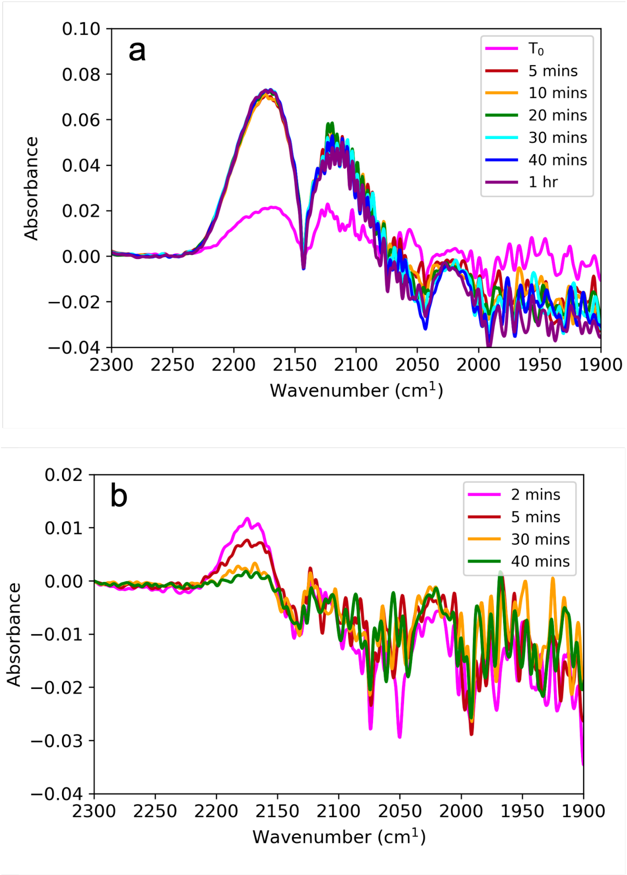

Figure S17. DRIFTS spectra of neat PDMS while a) flowing 100 sccm 10% CO in He and b) 100 sccm pure He following the CO dose.

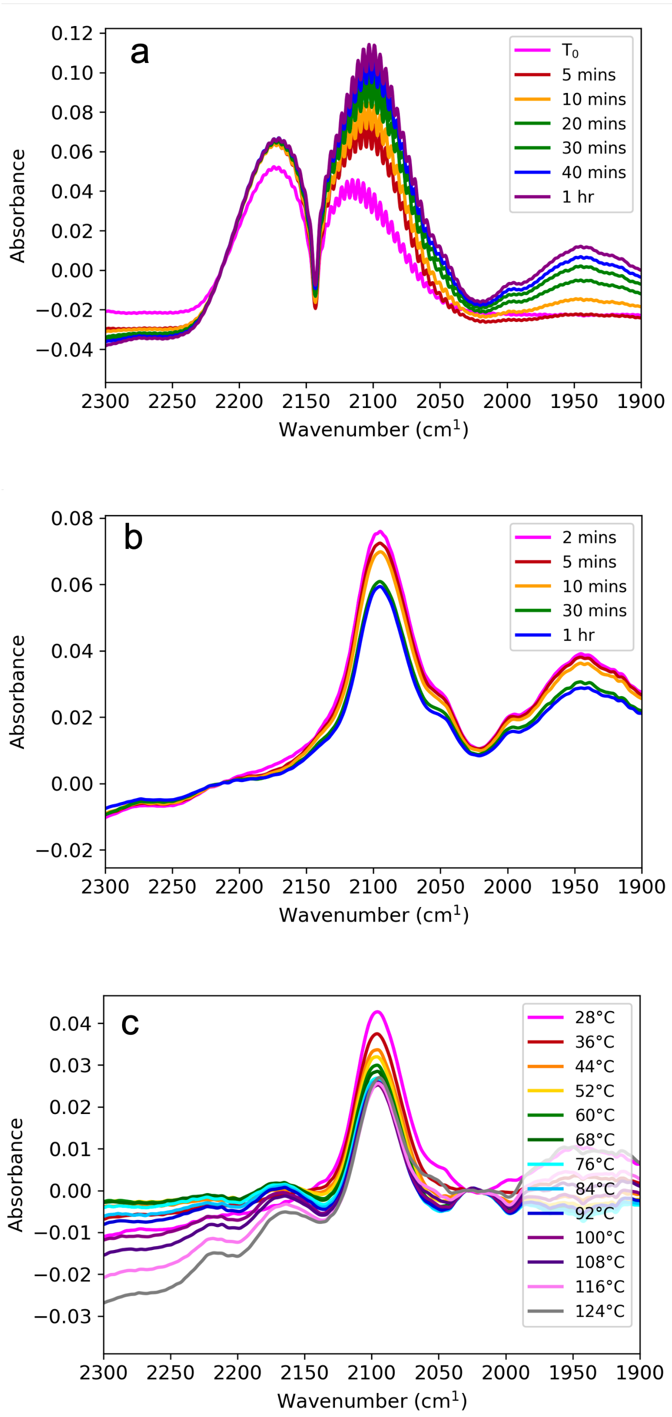

Figure S18. DRIFTS spectra of porous liquid made from 10 wt. % Cu(I)-COF@PDMS-MA in PDMS while a) flowing 100 sccm 10% CO in He, b) 100 sccm pure He following the CO dose, and c) heating at 10 °C/min under a He flow to follow CO desorption. Note, the background spectrum was collected at r.t., causing some baseline drift with heating.

X. Temperature Programmed Desorption

The ensuing discussion and experiments underscore the significance of both the thickness of the COF coating and the presence of a cross-linker in influencing H_2 sorption. In these experiments, samples were either dosed with H_2 at 77 K, or they were dosed at r.t. and then cooled to 77 K, prior to the vacuum and desorption cycle. The experiment is designed to probe whether the coating inhibits adsorption below its T_g. As can be seen in Fig. S19, the 'thick' coated material (with a 30 nm coating) does not adsorb any appreciable amount of H_2 during a 10 min dose at 77 K, unlike when it is dosed above its T_g. However, in the 'thin' coated sample (with a 5 nm coating), the material adsorbed ~10% of the H_2 it would otherwise adsorb when dosed above its T_g (green trace, Fig. S19). The results either suggest a fraction of the COF particles are not fully coated in the 'thin' sample, or that a small amount of H_2 can diffuse through a 5 nm coating during the 10 min. dose at 77 K.

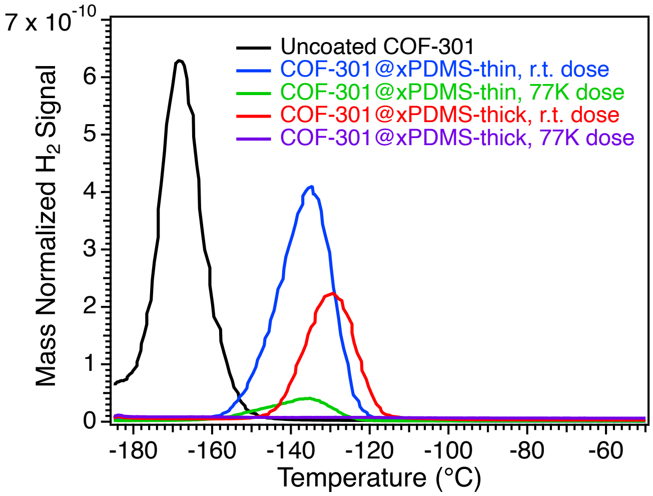

Figure S19. TPD measurements show H_2 desorption from uncoated COF-301 (black), COF-301@xPDMS-MA-thin dosed at r.t. (blue) and 77K (green), and COF-301@xPDMS-MA-thick dosed at r.t. (red) and 77K (purple). All signals were normalized to the total mass of the sample and the temperature was ramped at 15 °C/min.

Furthermore, the addition of the crosslinking agent appears instrumental in creating a barrier for H_2 diffusion. COF-301@PDMS-MA-'thick' (with no added crosslinker) readily adsorbed H_2 during a 10 min dose at 77 K (light blue trace, Fig. S20), similar to the uncoated material (black trace). The latter result suggests decreased segmental mobility in the polymer coating afforded by the crosslinker may strongly influence H_2 diffusion in these materials.

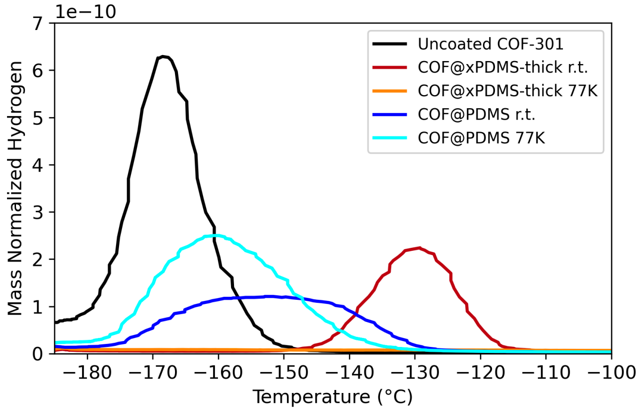

Figure S20. H_2 TPD traces for uncoated colloidal COF-301 (black), crosslinked COF-301@xPDMS-MA dosed with H_2 at r.t (red) and 77 K (orange), and non-crosslinked COF-301@PDMS-MA dosed with H_2 at r.t (dark blue) and 77 K (light blue).
